# The equation of state of the *Pmmn* phase of NiSi

**DOI:** 10.1107/S1600576715020087

**Published:** 2015-11-28

**Authors:** Oliver T. Lord, Andrew R. Thomson, Elizabeth T. H. Wann, Ian G. Wood, David P. Dobson, Lidunka Vocadlo

**Affiliations:** a School of Earth Sciences, University of Bristol, Wills Memorial Building, Queen’s Road, Bristol BS81RJ, UK; b Department of Earth Sciences, University College London, Gower Street, London WC1E 6BT, UK

**Keywords:** NiSi, equations of state, high pressure, diamond anvil cells

## Abstract

The equation of state of the orthorhombic phase of NiSi with *Pmmn* symmetry has been determined at room temperature from synchrotron-based X-ray diffraction measurements of its lattice parameters, made in a diamond anvil cell. Measurements were performed up to 44 GPa, using Ne as the pressure medium and Au as the pressure standard. The resulting pressure–volume (*P*–*V*) data have been fitted with a Birch–Murnaghan equation of state of third order to yield *V*_0_ = 11.650 (7) Å^3^ atom^−1^, *K*_0_ = 162 (3) GPa and *K*_0_′ = 4.6 (2). In addition, *P*–*V* data have been collected on Ni_53_Si_47_ in the B20 structure using both Ne and He as the pressure media and Cu and Au as the pressure standards, also to 44 GPa. A fit using the same Birch–Murnaghan equation of state of third order yields *V*_0_ = 11.364 (6) Å^3^ atom^−1^, *K*_0_ = 171 (4) GPa and *K*_0_′ = 5.5 (3).

## Abstract 2

The room-temperature equations of state of NiSi with *Pmmn* symmetry and Ni_53_Si_47_ in the B20 structure have been determined to 50 GPa using a diamond anvil cell. The equations of state are compared with existing experimental data and *ab initio* simulations.

## 1. Introduction   

Nickel monosilicide (NiSi), which crystallizes in the MnP (B31) structure (space group *Pnma*) at ambient pressure (Toman, 1951 ▸), has recently been shown to possess a surprisingly rich phase diagram (Lord *et al.*, 2014 ▸; Dobson *et al.*, 2015 ▸). Both the ∊-FeSi (B20) structure (space group *P*2_1_3) and the CsCl (B2) structure (space group *Pm*3*m*) were predicted to become stable at successively higher pressures on the basis of the static (0 K) *ab initio* computer simulations of Vočadlo *et al.* (2012 ▸). Both were subsequently detected experimentally (Lord *et al.*, 2012 ▸). Conversely, a new structure, with space group *Pmmn* was first detected in the run products of multi-anvil press (MAP) experiments quenched to room temperature from 1223 to 1310 K at 17.5 GPa and then recovered to atmospheric pressure. This new structure (hereafter referred to as *Pmmn*-NiSi), in which both the Ni and Si atoms have sixfold coordination, is essentially identical to the γ-CuTi structure type (space group *P*4/*mmn*) except that the *ab* plane of the unit cell is slightly distorted from square to rectangular; much more detail can be found in §6 of Wood *et al.* (2013 ▸). Subsequent static *ab initio* simulations have shown that this new structure has the lowest enthalpy at 0 K of all structures tested to date at pressure 21 < *P* < 264 GPa (Wood *et al.*, 2013 ▸). This new structure had been missed by previous experimental studies that employed laser heating in a diamond anvil cell (DAC) (Lord *et al.*, 2014 ▸, 2012 ▸) because the lowest temperature achieved in those studies was higher than the maximum extent of the stability field of *Pmmn*-NiSi (∼1200 K). It had also been missed by the previous *ab initio* study (Vočadlo *et al.*, 2012 ▸) simply because its rather unusual structure had not been considered.

The NiSi composition is of interest to us primarily because NiSi forms an end member in the Fe–Ni–Si ternary system, which encompasses compositions often employed as models for the cores of the terrestrial planets including the Earth. However, because many planetary bodies within our solar system have central pressures significantly lower than that of Earth (*e.g.* Mercury, ∼40 GPa, compared to Earth, ∼360 GPa), the low-pressure low-temperature parts of the Fe–Ni–Si system and its end-member constituents are important, including NiSi. For this reason we have studied this composition extensively in the past, including its phase diagram, across a wide range of pressures and temperatures (0–70 GPa and 500–3000 K) in both MAP and DAC experiments, and the equations of state (EoSs) of its constituent phases using both DAC experiments and *ab initio* simulations. In addition to its geophysical and planetary relevance, NiSi also has technological importance as a thin-film contact material in micro-electronics (*e.g.* Lavoie *et al.*, 2006 ▸). To date, we have *ab initio* EoSs available for all of the NiSi structures known to be stable, but only experimental EoSs for the B31, B20 and B2 structures. Here, we report the results of synchrotron-based powder X-ray diffraction measurements of the lattice parameters of *Pmmn*-NiSi in a DAC up to 44 GPa. In addition, we also provide new pressure–volume (*P*–*V*) data on slightly Ni enriched, non-stoichiometric NiSi in the B20 structure. The methods employed are described in §2 and the results are presented and discussed in §3, where they are also compared with the existing *ab initio* and experimental data.

## 2. Methods   

**Figure 1:**
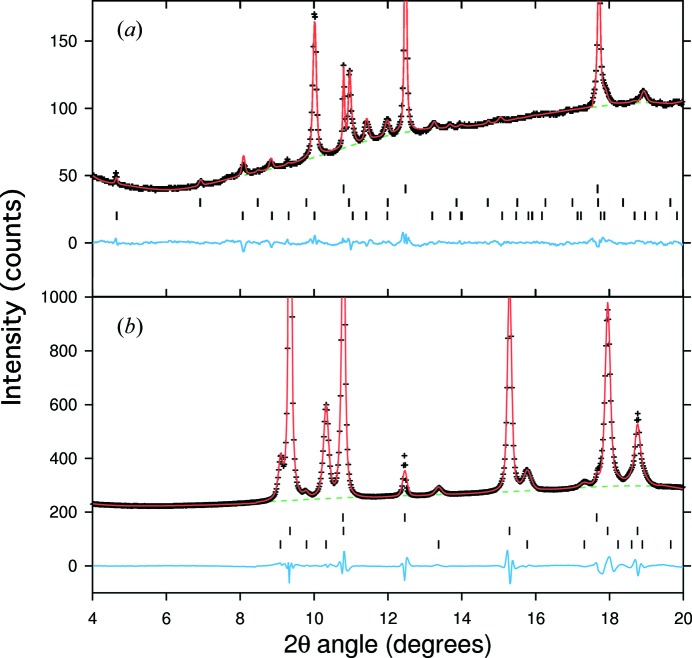
Examples of Le Bail fits (red lines), backgrounds (green dashed lines) and residuals (blue lines) for X-ray diffraction data (plus signs) collected in this study. Both patterns are at 17.7 (5) GPa. (*a*) The *Pmmn*-NiSi sample and (*b*) the Au standard. Tick marks represent reflections of, from top to bottom, fcc-Ne, B20-NiSi and *Pmmn*-NiSi in (*a*) and fcc-Ne, fcc-Au and hcp-Re (from the gasket; hcp denotes hexagonal close packed) in (*b*).

The starting materials for the two experiments reported here were selected from the crushed remains of the multi-anvil press synthesis experiment performed at 17.5 GPa and 1223 K described in §2.2 of Wood *et al.* (2013 ▸). This synthesis produced ∼80 vol.% of *Pmmn*-NiSi with a composition within error of the 1:1 NiSi stoichiometry (see §2.3 of Wood *et al.*, 2013 ▸) and ∼20 vol.% of material in the B20 (∊-FeSi) structure (space group *P*2_1_3) with a composition of Ni_53_Si_47_. As observed by Wood *et al.* (2013 ▸), the sample underwent slight back-transformation to the ambient-pressure B31 (MnP) structure (space group *Pnma*) during decompression. As a result, a trace of this phase is evident in our diffraction patterns, primarily as a broad feature at ∼7.8° (see Fig. 1 ▸ *a*), which we have not attempted to fit during our Le Bail refinements.

Pressure was generated using a membrane-driven Le Toullec type symmetric DAC with a 60° opening angle (Le Toullec *et al.*, 1988 ▸) and anvils of the Boehler–Almax design (Boehler & De Hantsetters, 2004 ▸), with culets of 300 µm diameter. Re gaskets were indented to a thickness of 40 µm and then a 150 µm-diameter hole was laser drilled in the centre of each of the indentations to form sample chambers. Into the sample chambers were loaded four spatially separated items, two of which were common to both experiments: a ruby sphere, used as a pressure marker, and a sample of B31-NiSi (for which the results will be presented in a future publication). In addition, experiment 1 contained a ∼25 µm-diameter polycrystalline grain of *Pmmn*-NiSi (with a trace of B20-Ni_53_Si_47_) and an Au pressure marker loaded as a loose polycrystalline aggregate, while experiment 2 contained a ∼25 µm-diameter polycrystalline grain of B20-Ni_53_Si_47_ and a Cu pressure marker, also loaded as a loose polycrystalline aggregate. The samples and pressure standards were separated so as to simplify the analysis of the X-ray diffraction patterns. The remaining space in the sample chambers was filled with a supercritical fluid pressure transmitting medium of Ne (experiment 1) or He (experiment 2), using the high-pressure loading system at the European Synchrotron Radiation Facility (ESRF). The cells were then sealed, with an initial pressure of ∼0.2 GPa as determined by ruby fluorescence spectroscopy. Ne, which solidifies at 4.8 GPa at 300 K, has been shown to remain essentially hydrostatic up to 15 GPa (Klotz *et al.*, 2009 ▸). Even at 50 GPa (which encompasses this study), the degree of non-hydrostaticity of solid Ne is minor, supporting pressure gradients of only ∼0.5 GPa (*i.e.* 1%; Klotz *et al.*, 2009 ▸). He, which solidifies at 12.1 GPa at 300 K and remains essentially hydrostatic up to 20 GPa, is even more effective, supporting pressure gradients of only ∼0.15 GPa at 50 GPa (*i.e.* 0.3%; Klotz *et al.*, 2009 ▸). Note that the use of laser annealing to reduce deviatoric stress after each pressure step was not possible in this study: below ∼12 GPa, both samples would convert, upon heating, to B31-NiSi. Above ∼12 GPa, temperatures amenable to measurement by spectroradiometry (>1200 K) risk pushing the *Pmmn*-NiSi sample further into the two-phase *Pmmn*-NiSi + B20-NiSi region of the phase diagram, altering the stoichiometry of the *Pmmn*-NiSi phase, or even converting it entirely to the B20-NiSi structure (Dobson *et al.*, 2015 ▸). Laser annealing of the B20-Ni_53_Si_47_ sample was not possible either, owing to the proximity to the B31-NiSi sample, which would convert above 12 GPa to *Pmmn*-NiSi, B20-NiSi or both, depending on temperature.

The samples were compressed by incrementally increasing the pressure in the membrane of the DAC using an automatic pressure controller. At each step, after waiting for ∼5 min to allow the gasket and sample to relax under the increased load, separate X-ray diffraction patterns were collected, one from each of the two samples and one from the pressure standard (either Au or Cu). X-ray powder diffraction was performed at beamline ID27 of the ESRF (Mezouar *et al.*, 2005 ▸) using a monochromatic beam with an energy of 33 keV (λ = 0.3738 Å). Diffracted X-rays were collected using a MAR165 CCD detector at a distance from the sample of ∼260 mm, calibrated exactly using an LaB_6_ standard. The resulting two-dimensional patterns were integrated into one-dimensional spectra using the *Fit2D* program (Hammersley, 1997 ▸) and then fitted using the Le Bail method (Le Bail *et al.*, 1988 ▸) as implemented in the *GSAS* suite of programs (Larson & Von Dreele, 1994 ▸; Toby, 2001 ▸).

## 3. Results and discussion   

### 3.1. Calibration of pressure   

In this study, we have opted to use the Au and Cu standards, rather than the Ne pressure medium, as our pressure calibrants because we believe them to be more accurate. This is primarily because Ne (*K*_0_ ≃ 1) is so much more compressible than Au (*K*_0_ ≃ 170) that small differences in the parameters of the Ne EoS result in significant differences in the calculated pressure. So, while the EoS parameters for Ne reported in the two most recent studies, those of Dewaele *et al.* (2008 ▸) and Dorfman *et al.* (2012 ▸), are in close agreement (*K*_0_ = 1.07 GPa *versus* 1.04 GPa and *K*_0_′ = 8.4 *versus* 8.48), they nevertheless result in significant differences in the calculated pressures (Table 1 ▸). The EoSs of Au and Cu do not suffer from this problem, and, for Au, there is consensus between recent EoS studies (Dewaele *et al.*, 2004 ▸; Fei *et al.*, 2007 ▸). The EoSs for Au and Cu employed here (Dewaele *et al.*, 2004 ▸) were determined from samples loaded together in the same noble gas pressure medium as was used in the present study; they diverge from each other by <1 GPa at 50 GPa and should be directly applicable to our experimental design. Another disadvantage of using Ne as the pressure calibrant, especially when laser annealing is not possible (see §2), is that it tends to show a tetragonal distortion under uniaxial compression, making it impossible to accurately fit its Bragg reflections using a face-centred cubic (fcc) unit cell (Fig. 1 ▸ *a*). Such distortions are less prominent in the Au and Cu pressure standards (Fig. 1 ▸ *b*), possibly because the standards are free floating within the Ne or He medium and thus experience quasi-hydrostatic conditions, whereas the Ne bridges the diamond anvils and is therefore subject to significant deviatoric stress. Finally, because Ne does not crystallize until 4.8 GPa at 300 K it cannot be used as a calibrant below this pressure. He scatters X-rays so weakly that it cannot be used as a standard even above its 300 K solidification pressure of 12.1 GPa.

### 3.2. The equation of state of *Pmmn*-NiSi (experiment 1)   

Table 2 ▸ compares the lattice parameters of *Pmmn*-NiSi at ambient pressure as measured in this study with the measurements and *ab initio* simulations of Wood *et al.* (2013 ▸). The two sets of experimental measurements are almost identical. The *ab initio* simulations, however, indicate much more significant differences when compared with the experiments, with all three axes being longer, resulting in a volume that is 1.3% larger than the experimental value in this study. This difference is, however, in line with the overestimation of volume of around 1% common in *ab initio* simulations that employ the generalized gradient approximation (GGA) as was used by Wood *et al.* (2013 ▸); similar discrepancies between DAC experiments and GGA-based *ab initio* simulations of 1 and 1.4% were observed for B31-NiSi and B20-NiSi, respectively (Lord *et al.*, 2012 ▸; Vočadlo *et al.*, 2012 ▸).

**Figure 2:**
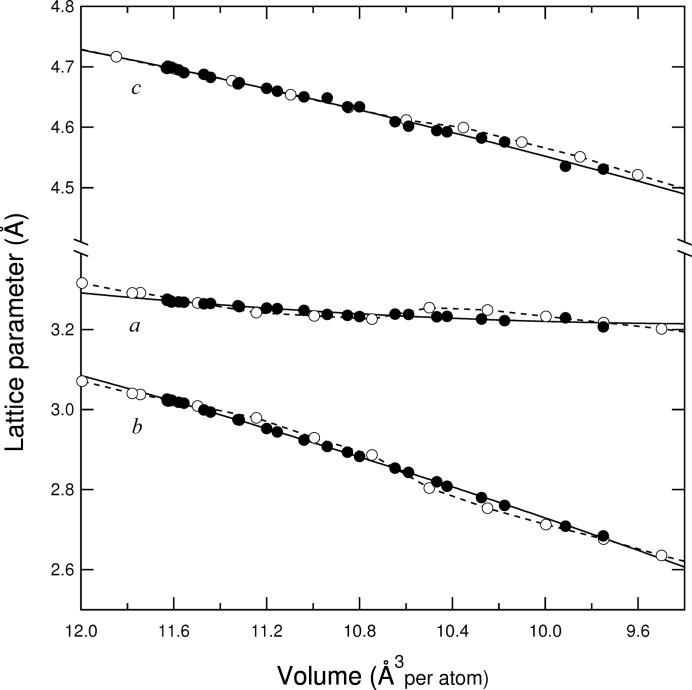
Lattice parameters of *Pmmn*-NiSi as a function of unit-cell volume from this study (black circles) and the *ab initio* simulations of Wood *et al.* (2013 ▸; open circles with dashed lines). The solid lines are polynomial fits of second order to the experimental data collected in this study. Note the break in the *y* axis at *y* ≃ 3.4. The kink in the *ab initio* lattice parameters can be seen much more clearly in Fig. 4(*a*) of Wood *et al.* (2013 ▸).

The lattice parameters of *Pmmn*-NiSi from Table 1 ▸ are presented as a function of volume in Fig. 2 ▸, along with the results of the *ab initio* simulations of Wood *et al.* (2013 ▸). Those simulations show a pronounced kink in all three lattice parameters (though it is most prominent in *a* and *b*), at 10.5 < *V <* 10.8 Å^3^ atom^−1^, such that the *a* axis actually lengthens over a short interval of compression. In comparison, our experimental data only show such a kink on the *a* axis, though it is not statistically significant; the *b* and *c* axes shorten smoothly over the investigated compression range and all three can be well described with a polynomial function of second order.

**Figure 3:**
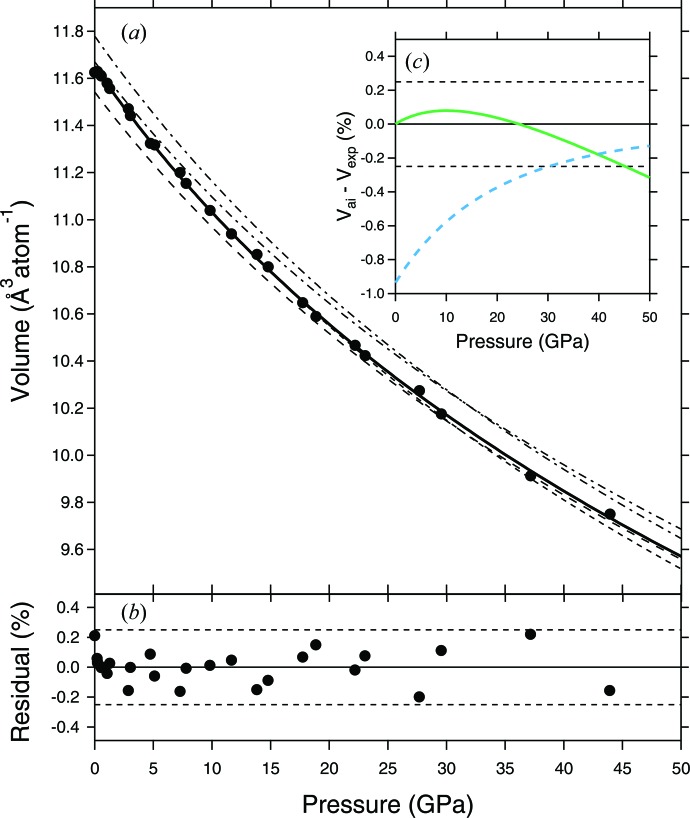
(*a*) Volume per atom (Å^3^) for *Pmmn*-NiSi as a function of pressure as calculated from the Au pressure marker using the EoS of Dewaele *et al.* (2004 ▸; filled circles). The solid line is a third-order Birch–Murnaghan EoS fitted to the data with all three parameters allowed to vary (*V*_0_, *K*_0_ and *K*_0_′). The dashed lines are the volume-corrected EoS fitted to the *ab initio* simulation results of Wood *et al.* (2013 ▸) over the range 10.75 < *V* < 12.0 Å^3^ atom^−1^ (short dashes) and 6.5 < *V* < 10.5 Å^3^ atom^−1^ (long dashes). The uncorrected *ab initio* EoSs are represented by the dash–dot lines; see text for details. (*b*) Residuals of the fit to the data based on the Au standard in which *V*_0_ was allowed to vary. The thin dashed lines represent ±0.25% *V*. (*c*) *V*_ai_ − *V*_exp_ for the low-pressure (green thick solid line) and high-pressure (blue thick dashed line) *ab initio* EoSs (both volume corrected). The thin solid line at *y* = 0 represents the experimental EoS presented in (*a*) and the thin dashed lines represent ±0.25% *V* as in (*b*).

The smooth change in the lattice parameters as a function of pressure makes it reasonable to fit all of the compression data using a single EoS, rather than fitting the data separately either side of *V* ≃ 10.6 Å^3^ atom^−1^ as Wood *et al.* (2013 ▸) did. The results of our preferred third-order Birch–Murnaghan fit, in which all three parameters (*V*_0_, *K*_0_ and *K*_0_′) were allowed to vary, is shown in Fig. 3 ▸(*a*) as the solid black line; the fitted parameters are presented in Table 3 ▸. As can be seen in Fig. 3 ▸(*b*), all of the data fall within ±0.25% *V* of the fitted curve and appear randomly distributed around the 0% line; the average mismatch is just 0.08% *V*. Also plotted in Fig. 3 ▸(*a*), as dash–dot lines, are the high- and low-pressure EoSs from Wood *et al.* (2013 ▸). To facilitate comparison between the experimental and *ab initio* results, we have corrected the low-pressure *ab initio* EoS by applying a constant volume offset of −0.129 Å^3^ atom^−1^ (−1.1%) such that its *V*_0_ is equal to the measured value of 11.650 Å^3^ atom^−1^ (the long-dashed line). The same relative offset of −1.1% has been applied to the high-pressure *ab initio* EoS (the short-dashed line). Though we do not see the ‘kink’ in the lattice parameters that is clearly apparent in Fig. 4(*a*) of Wood *et al.* (2013 ▸), it is apparent from Fig. 3 ▸(*a*) that their volume-corrected low-pressure EoS matches the data slightly better below ∼25 GPa, while their volume-corrected high-pressure EoS matches the data better at higher pressures. This can be seen more clearly from the variation with pressure in the difference between the volume-corrected and experimental *ab initio* EoSs (represented by *V*_ai_ − *V*_exp_; Fig. 3 ▸ *c*). This observation might suggest that a continuous transition does occur in the *Pmmn*-NiSi structure, but that it is less pronounced at 300 K (the temperature at which the experiments are performed) than it is at 0 K (the temperature at which the simulations are performed).

It is clear from Table 2 ▸ that the values for *K*_0_ and *K*_0_′ from our experimental EoS are reasonably close to the values determined from the fits to the *ab initio* simulations of Wood *et al.* (2013 ▸). This is in spite of the fact that the range of compression achieved in the experiments is much smaller than that achieved in the simulations, and that *K*_0_ and *K*_0_′ have a strong negative correlation coefficient of −0.95.

### 3.3. The equation of state of B20-structured Ni_53_Si_47_ (experiments 1 and 2)   

**Figure 4:**
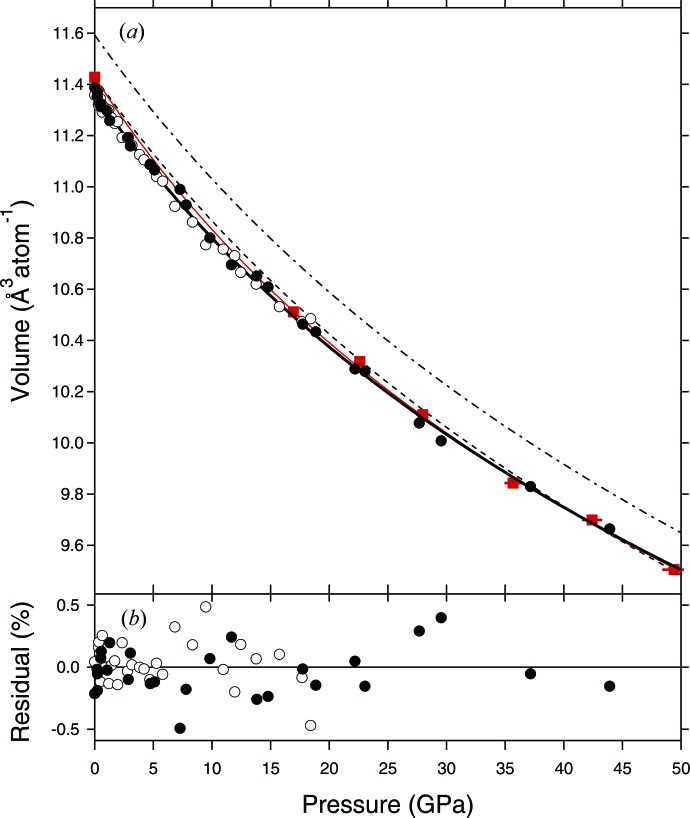
Volume per atom for B20-Ni_53_Si_47_ as a function of pressure as calculated from the Cu pressure marker using the EoS of Dewaele *et al.* (2004 ▸; circles). The filled circles are from experiment 1 (in Ne) and the open circles from experiment 2 (in He). The thick solid line is a third-order Burch–Murnaghan EoS fitted to all the data (Table 2 ▸). The dashed line is the EoS fitted to the *ab initio* simulations from Vočadlo *et al.* (2012 ▸), which has been corrected so that its *V*_0_ is equal to that of the experimentally determined value. The uncorrected *ab initio* EoS is represented by the dash–dot line. The thin solid red line is the EoS fitted to the *P*–*V* data of Lord *et al.* (2012 ▸; red squares), which was produced using NaCl as the pressure medium coupled with laser annealing on a stoichiometric NiSi sample.

The *P*–*V* data for B20-Ni_53_Si_47_ from experiment 1 are presented in Table 1 ▸, while the data from experiment 2 are presented in Table 4 ▸. All the data are plotted in Fig. 4 ▸ as a function of pressure, together with a third-order Birch–Murnaghan fit to the two data sets combined, in which all three fitted parameters were allowed to vary (Table 3 ▸). The two data sets are in excellent agreement with each other, which is a reflection of the facts that, firstly, there is little difference between Ne and He as a pressure medium at pressures up to ∼50 GPa and that, secondly, the Cu and Au EoSs are themselves in very close agreement. The new data are also in excellent agreement with the data from the paper by Lord *et al.* (2012 ▸) in which NaCl was used as the pressure medium and pressure calibrant and laser annealing was employed after each compression step to reduce deviatoric stress. This suggests that laser annealing of samples contained in significantly non-hydrostatic pressure media, such as NaCl, is at least as effective at minimizing deviatoric stress as the use of quasi-hydrostatic media such as He and Ne without laser annealing. This also indicates that the EoS of Cu from Dewaele *et al.* (2004 ▸) used in this study and the EoS of NaCl in either the B1 (Dorogokupets & Dewaele, 2007 ▸; below 30 GPa) or B2 structures (Fei *et al.*, 2007 ▸; above 30 GPa) used by Lord *et al.* (2012 ▸) must be in good agreement over the pressure range of this study.

Comparing the fitted values of *K*_0_ and *K*_0_′ from this study with those from Lord *et al.* (2012 ▸) indicates that the non-stoichiometric Ni-rich material studied here is somewhat stiffer at ambient pressure than stoichiometric B20-NiSi, while *V*_0_ (Table 3 ▸) is ∼0.6% smaller. This difference is significant, given the <0.1% difference in the volume of *Pmmn*-NiSi measured in this study as compared to that of Wood *et al.* (2013 ▸), and is probably due to the slight Ni enrichment of the sample relative to the near stoichiometric sample used by Lord *et al.* (2012 ▸). As is the case for *Pmmn*-NiSi, the *ab initio* EoS for stoichiometric B20-NiSi from Vočadlo *et al.* (2012 ▸) [represented by the dash–dot line in Fig. 4 ▸(*a*)] has a significantly larger *V*_0_ than the experimentally determined value (about 1.4% larger; see Table 3 ▸). As before, we have decided to correct the *ab initio* EoS by applying a constant volume offset of −0.1641 Å^3^ atom^−1^ such that its *V*_0_ is equal to the value measured for stoichiometric B20-NiSi by Lord *et al.* (2012 ▸) of 11.4289 Å^3^ atom^−1^ [the long-dashed line in Fig. 4 ▸(*a*)]. This corrected *ab initio* EoS for B20-NiSi matches closely all of the experimental data over this pressure range. However, above 50 GPa (not shown in the figure) the *ab initio* and experimental EoSs diverge, with the former being more compressible, yielding smaller volumes at a given pressure. This is a consequence of *K*_0_′ from the *ab initio* EoS being significantly smaller than the experimentally determined values (Table 3 ▸). The corollary of this is that the uncorrected *ab initio* EoS, which initially overestimates volume, crosses the extrapolated experimental EoS at ∼150 GPa (see Fig. 5 of Lord *et al.*, 2012 ▸). However, compression data for B20-NiSi are only available to 80 GPa; were data available to higher pressure it would be interesting to see whether the uncorrected *ab initio* and experimental EoSs would in fact cross as extrapolation predicts or, alternatively, converge. If the latter, this would suggest that the *ab initio* simulations, on this material at least, do a better job of predicting the correct volume at higher pressures. This would also mean that ‘correcting’ an *ab initio* EoS such that its *V*_0_ matched that of its experimental counterpart would not necessarily be a valid approach.

### 3.4. Comparison with other NiSi structures   

**Figure 5:**
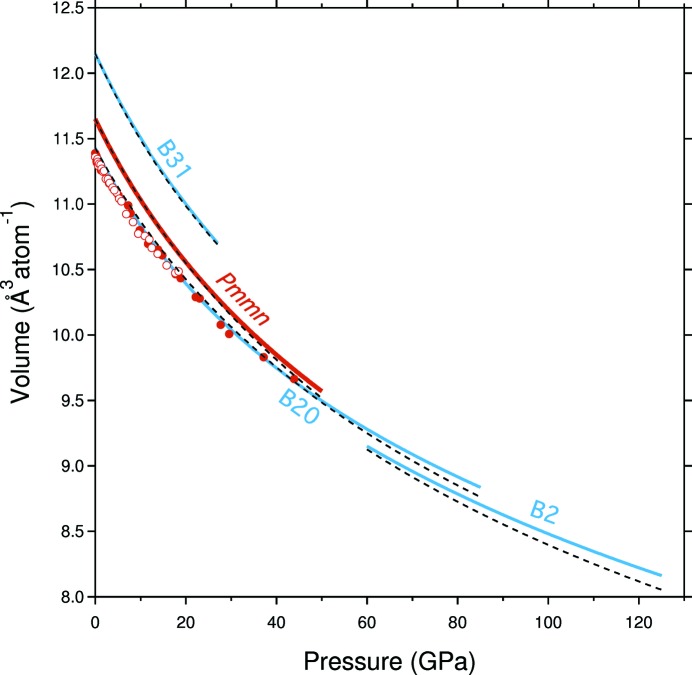
Comparison of the experimentally determined *P*–*V* curves for the polymorphs of NiSi measured to date. The EoSs of B31-, B20- and B2-NiSi are from Lord *et al.* (2012 ▸; thin blue lines), while the EoS of *Pmmn*-NiSi is from this study (thick red line). The circles represent the *P*–*V* data for B20-NiSi from experiments 1 (filled) and 2 (open) of this study. The dashed lines represent the *ab initio* EoSs from Vočadlo *et al.* (2012 ▸) for B31-, B20- and B2-NiSi; the EoS for *Pmmn*-NiSi is from Wood *et al.* (2013 ▸) and is the average of their low-pressure and high-pressure EoSs. A constant volume offset has been applied to all the *ab initio* EoSs so that their *V*_0_ values match the relevant measured or estimated experimental values.

Fig. 5 ▸ is a summary of the ambient-temperature *P*–*V* curves of the polymorphs of NiSi determined to date, including B31-NiSi, B20-NiSi and B2-NiSi results from Lord *et al.* (2012 ▸) and *Pmmn*-NiSi and B20-Ni_53_Si_47_ from this study. These four phases represent all of the constituents of the part of the NiSi phase diagram of relevance to planetary interiors (Lord *et al.*, 2014 ▸, 2012 ▸; Dobson *et al.*, 2015 ▸). At 300 K, the expected sequence of phases with increasing pressure is B31 → *Pmmn* → B20 → B2. As expected, this sequence is one of decreasing *V*_0_, increasing coordination number and increasing symmetry. Further, while neither *K*_0_ nor *K*_0_′ show the expected monotonic increase with increasing stabilization pressure, the product of the two parameters does (B31 = 660 GPa, *Pmmn* = 745 GPa, B20 = 902 GPa and B2 = 920 GPa). This is a manifestation of the high degree of correlation between these two fitted parameters within the Birch–Murnaghan formalism (see §3.2).

Included in Fig. 5 ▸ are the corresponding *ab initio* EoSs (as dashed lines) from Vočadlo *et al.* (2012 ▸) for B31-NiSi, B20-NiSi and B2-NiSi and from Wood *et al.* (2013 ▸) for *Pmmn*-NiSi, corrected such that *V*_0_ matches the experimentally determined value. In the case of the unrecoverable B2 phase, *V*_0_ cannot be measured, and so a correction of the same magnitude as found for the B20 phase, of −1.4%, has been applied. In the case of the *Pmmn* phase, the plotted curve represents the average of the low-pressure and high-pressure *ab initio* EoSs, after each has been volume corrected. It is apparent from this analysis that, despite the subtle differences described in the previous sections, the volume-corrected *ab initio* EoSs match the majority of the experimental data rather well, and within error at all conditions for which experimental data exist. This is impressive given the range of compression and symmetry involved. It is only above ∼80 GPa that significant divergence starts to be apparent, as might be expected for extrapolations beyond the pressure range over which the data were collected.

## 4. Conclusion   

Room-temperature EoSs for Pmmn-NiSi and Ni_53_Si_47_ in the B20 structure have been determined experimentally up to 44 GPa from X-ray diffraction measurements in a DAC. In both cases, the new data corroborate previous experimental measurements from Wood *et al.* (2013 ▸) for *Pmmn*-NiSi at ambient pressure and Lord *et al.* (2012 ▸) for B20-NiSi at high pressure. There is also good agreement with the *ab initio P*–*V* data of Wood *et al.* (2013 ▸) for *Pmmn*-NiSi and Vočadlo *et al.* (2012 ▸) for B20-NiSi, once a constant volume offset has been applied to the *ab initio* results such that the experimental and *ab initio* values for *V*_0_ are equal. Such a correction is considered valid given that GGA-based *ab initio* simulations commonly overestimate volume (at least at ambient pressure) by ∼1%. However, we see no strong evidence for the subtle second-order structural transition observed by Wood *et al.* (2013 ▸) in the lattice parameters of *Pmmn*-NiSi at 10.5 < *V <* 10.8 Å^3^ atom^−1^ in their simulations. As a result of this study, experimentally determined ambient-temperature EoSs for all of the constituents of that part of the NiSi phase diagram relevant to planetary interiors are now available.

## Supplementary Material

Crystal structure: contains datablock(s) global, OTL110213ESRF53__001_publ. DOI: 10.1107/S1600576715020087/kc5022sup1.cif

Crystal structure: contains datablock(s) global, OTL110213ESRF53__030_publ, OTL110213ESRF53__030_overall, OTL110213ESRF53__030_phase_1, OTL110213ESRF53__030_phase_2, OTL110213ESRF53__030_p_01. DOI: 10.1107/S1600576715020087/kc5022sup2.cif

## Figures and Tables

**Table 1:** Compression data for experiment 1

			*Pmmn*-NiSi				B20-NiSi	
*P*_Au_ (GPa)†	*P*_Ne_ (GPa)‡	*P*_Ne_ (GPa)§	*a* (Å)	*b* (Å)	*c* (Å)	*V_Pmmn_* (Å^3^ atom^−1^)	*a* (Å)	*V*_B20_ (Å^3^ atom^−1^)
0 (0)	–	–	3.2742 (8)	3.021 (1)	4.701 (1)	11.626 (3)	2.25 (2)	11.388 (8)
0.20 (1)	–	–	3.2725 (6)	3.0260 (4)	4.697 (1)	11.629 (3)	2.248 (2)	11.353 (8)
0.24 (1)	–	–	3.2755 (7)	3.0222 (6)	4.699 (1)	11.629 (3)	2.25 (1)	11.37 (6)
0.24 (1)	–	–	3.2733 (8)	3.0238 (6)	4.6998 (9)	11.629 (22)	2.248 (5)	11.35 (3)
0.51 (1)	–	–	3.2714 (8)	3.0223 (6)	4.698 (1)	11.613 (3)	2.245 (3)	11.32 (2)
0.56 (2)	–	–	3.2685 (8)	3.0235 (7)	4.699 (1)	11.610 (3)	2.245 (3)	11.31 (2)
1.06 (3)	–	–	3.2691 (9)	3.018 (7)	4.695 (1)	11.580 (3)	2.244 (1)	11.298 (6)
1.28 (4)	–	–	3.268 (1)	3.0158 (8)	4.690 (1)	11.557 (3)	2.2413 (9)	11.259 (4)
2.87 (7)	–	–	3.264 (1)	2.999 (1)	4.688 (1)	11.471 (3)	2.237 (4)	11.194 (2)
3.04 (8)	–	–	3.265 (1)	2.9937 (7)	4.683 (1)	11.442 (3)	2.2347 (3)	11.160 (1)
4.7 (1)	–	–	3.259 (1)	2.9748 (9)	4.672 (2)	11.324 (4)	2.2299 (8)	11.089 (4)
5.1 (1)	–	–	3.257 (1)	2.9739 (9)	4.674 (1)	11.318 (3)	2.228 (2)	11.066 (8)
7.3 (2)	6.77 (5)	5.6 (1)	3.2533 (7)	2.9524 (6)	4.664 (1)	11.201 (3)	2.223 (2)	10.990 (9)
7.8 (2)	7.69 (5)	6.4 (1)	3.2521 (6)	2.9443 (5)	4.660 (1)	11.154 (3)	2.219 (2)	10.930 (9)
9.8 (2)	10.17 (7)	8.5 (1)	3.248 (2)	2.924 (1)	4.650 (4)	11.039 (9)	2.21 (2)	10.80 (1)
11.7 (3)	12.59 (8)	10.6 (2)	3.237 (3)	2.908 (3)	4.648 (4)	10.94 (1)	2.203 (1)	10.696 (6)
13.8 (4)	14.26 (9)	12.1 (2)	3.235 (4)	2.894 (3)	4.634 (5)	10.85 (1)	2.2 (3)	10.65 (1)
14.8 (4)	15.6 (1)	13.2 (2)	3.232 (3)	2.883 (3)	4.634 (6)	10.80 (1)	2.197 (4)	10.61 (2)
17.7 (5)	18.5 (1)	15.9 (3)	3.2383 (5)	2.8535 (4)	4.609 (1)	10.648 (3)	2.1873 (1)	10.464 (1)
18.9 (5)	19.9 (1)	17.1 (3)	3.238 (1)	2.8431 (9)	4.602 (2)	10.589 (4)	2.1851 (5)	10.434 (3)
22.2 (5)	23.3 (2)	20.1 (3)	3.232 (1)	2.8197 (9)	4.594 (2)	10.467 (4)	2.175 (6)	10.289 (3)
23.0 (6)	24.8 (2)	21.5 (4)	3.233 (2)	2.809 (1)	4.593 (3)	10.423 (6)	2.1743 (9)	10.279 (4)
27.7 (7)	29.4 (2)	25.7 (4)	3.227 (3)	2.780 (2)	4.582 (3)	10.275 (6)	2.16 (6)	10.078 (3)
29.5 (8)	32.8 (2)	28.8 (5)	3.222 (3)	2.761 (2)	4.576 (3)	10.176 (7)	2.155 (6)	10.008 (3)
37.2 (9)	41.7 (3)	36.8 (6)	3.229 (2)	2.709 (2)	4.535 (3)	9.913 (6)	2.13 (7)	9.830 (3)
44 (1)	47.4 (3)	42.1 (7)	3.2066 (8)	2.6845 (8)	4.531 (6)	9.75 (1)	2.1421 (7)	9.664 (3)

† Based on the EoS of Dewaele *et al.* (2004 ▸).

‡ Based on the EoS of Dewaele *et al.* (2008 ▸).

§ Based on the EoS of Dorfman *et al.* (2012 ▸).

**Table 2:** Lattice parameters for *Pmmn*-NiSi at ambient pressure

		Wood *et al.* (2013 ▸)†			
	This Study	Experimental		*Ab initio*	
*a* (Å)	3.2742 (8)	3.2735 (1)	−0.02%	3.2911	0.52%
*b* (Å)	3.021 (1)	3.0266 (1)	0.19%	3.0404	0.64%
*c* (Å)	4.701 (1)	4.69776 (6)	−0.07%	4.7088	0.17%
*V* (Å^3^ atom^−1^)	11.626 (3)	11.6360 (3)	0.09%	11.7793	1.32%

† Percentages represent differences relative to values measured in this study.

**Table 3:** Equation of state fitting parameters

	*Pmmn*-NiSi			B20-NiSi		
	*V*_0_ (Å^3^ atom^−1^)	*K*_0_ (GPa)	*K*_0_′	*V*_0_ (Å^3^ atom^−1^)	*K*_0_ (GPa)	*K*_0_′
This Study						
9.8 < *V* < 11.6	11.650 (7)	162 (3)	4.6 (2)	11.364 (6)	171 (4)	5.5 (3)

Wood *et al.* (2013 ▸)						
10.75 < *V* < 12.0	11.7793 (5)	166.826 (3)	4.05 (8)	–	–	–
6.5 < *V* < 10.5	11.670 (6)	175.563 (5)	4.348 (8)	–	–	–

Lord *et al.* (2012 ▸)						
–	–	–	–	11.4289†	161 (3)	5.6 (2)

Vočadlo *et al.* (2012 ▸)						
–	–	–	–	11.593 (3)	180.143 (4)	4.48 (1)

† Fixed at the value measured by Lord *et al.* (2012 ▸).

**Table 4:** Compression data for experiment 2

	B20-NiSi		
*P*_Cu_ (GPa)†	*a* (Å)	*V* (Å^3^ atom^−1^)	
0	2.2479	11.359 (1)	
0.32 (2)	2.2456 (1)	11.324 (1)	
0.36 (2)	2.2452 (2)	11.317 (1)	
0.39 (2)	2.2465 (2)	11.336 (1)	
0.44 (2)	2.246 (2)	11.322 (1)	
0.49 (2)	2.2470 (2)	11.344 (1)	
0.57 (2)	2.2456 (3)	11.323 (2)	
0.65 (2)	2.2435 (3)	11.292 (1)	
0.85 (2)	2.2446 (3)	11.309 (2)	
1.2 (2)	2.2441 (2)	11.301 (1)	
1.42 (2)	2.2421 (1)	11.270 (1)	
1.71 (2)	2.2406 (2)	11.248 (1)	
1.95 (2)	2.2410 (2)	11.255 (1)	
2.34 (2)	2.237 (1)	11.193 (1)	
2.8 (2)	2.2368 (1)	11.191 (1)	
3.18 (2)	2.2349 (1)	11.162 (1)	
3.83 (4)	2.2325 (2)	11.126 (1)	
4.2 (2)	2.2311 (2)	11.106 (1)	
4.70 (2)	2.2299 (1)	11.087 (1)	
5.26 (3)	2.2268 (1)	11.042 (1)	
5.79 (4)	2.2255 (3)	11.022 (1)	
6.84 (3)	2.2189 (1)	10.924 (1)	
8.34 (3)	2.2147 (2)	10.863 (1)	
9.47 (5)	2.2086 (2)	10.774 (1)	
10.95 (3)	2.2075 (1)	10.757 (1)	
11.94 (3)	2.2057 (3)	10.731 (1)	
12.46 (6)	2.2012 (3)	10.666 (1)	
13.77 (6)	2.1981 (2)	10.620 (1)	
15.75 (6)	2.192 (1)	10.532 (1)	
17.67 (6)	2.1879 (4)	10.474 (2)	
18.41 (6)	2.1887 (2)	10.485 (1)	

† Based on the EoS of Dewaele *et al.* (2004 ▸).
